# Embedding Biometric Information in Interpolated Medical Images with a Reversible and Adaptive Strategy

**DOI:** 10.3390/s22207942

**Published:** 2022-10-18

**Authors:** Heng-Xiao Chi, Ji-Hwei Horng, Chin-Chen Chang, Yung-Hui Li

**Affiliations:** 1 Department of Information Engineering and Computer Science, Feng Chia University, Taichung 40724, Taiwan; 2 Department of Electronic Engineering, National Quemoy University, Kinmen 89250, Taiwan; 3 AI Research Center, Hon Hai (Foxconn) Research Institute, Taipei City 114699, Taiwan

**Keywords:** data hiding, interpolation, reversible data hiding, medical image

## Abstract

How to hide messages in digital images so that messages cannot be discovered and tampered with is a compelling topic in the research area of cybersecurity. The interpolation-based reversible data hiding (RDH) scheme is especially useful for the application of medical image management. The biometric information of patients acquired by biosensors is embedded into an interpolated medical image for the purpose of authentication. The proposed scheme classifies pixel blocks into complex and smooth ones according to each block’s dynamic range of pixel values. For a complex block, the minimum-neighbor (MN) interpolation followed by DIM embedding is applied, where DIM denotes the difference between the block’s interpolated pixel values and the maximum pixel values. For a smooth block, the block mean (BM) interpolation is followed by a prediction error histogram (PEH) embedding and a difference expansion (DE) embedding is applied. Compared with previous methods, this adaptive strategy ensures low distortion due to embedding for smooth blocks while it provides a good payload for complex blocks. Our scheme is suitable for both medical and general images. Experimental results confirm the effectiveness of the proposed scheme. Performance comparisons with state-of-the-art schemes are also given. The peak signal to noise ratio (PSNR) of the proposed scheme is 10.32 dB higher than the relevant works in the best case.

## 1. Introduction

Hiding information that does not want to be discovered or tampered with in digital image carriers is a hot issue in the research area of information security. According to the application purpose, it can be divided into steganography and watermarking. Typically, people use watermarking techniques for copyright protection and steganography for covert communication. Data hiding methods can also be divided into two kinds: reversible and irreversible, depending on the receiver’s ability to recover the cover digital image. Reversible data hiding (RDH) [1,2,3,4,5,6,7,8,9,10,11,12,13] can recover the primary digital image after reading the embedded secret messages. While irreversible data hiding (IRDH) [14,15] cannot recover the primary digital image from the message-embedded confidential image completely without loss. Nevertheless, the IRDH method is usually able to embed more messages than the RDH method.

Recently, many RDH schemes based on the interpolated cover image were proposed [16,17,18,19,20,21,22,23,24,25,26,27,28,29,30], which can embed more secret data than traditional RDH methods while preserving a good visual quality of confidential images. The interpolation-based RDH scheme is especially useful for the application of medical image management. Since the visual quality of clinical images is directly related to the diagnosis, perceptible distortion is unacceptable. Therefore, it is more desirable to keep the original pixel value unchanged. The cover image is usually generated by upsampling with interpolation technology in the interpolation-based RDH method. Then, the confidential message is hidden into the interpolated pixels only. In this way, after extracting the confidential data, we only need to delete the interpolated pixels from the confidential image, and we can recover the primary image perfectly.

In e-healthcare information management, if we can store and transmit the biometric information of a patient together with its corresponding medical image, an authentication procedure can be executed to ensure correct usage of the medical image. Moreover, we need to hide the information in its corresponding medical image with the least distortion to prevent misdiagnosis. The interpolation-based RDH method provides a good solution for this demand.

The key techniques for interpolation-based RDH are the interpolation method and the data embedding method. Previous work in interpolation technology includes the neighbor mean interpolation (NMI) [16,17,18], the interpolation by neighboring pixel (INP) [19], and the pixel repetition technology, etc. In 2021, Mandal et al. [20] published an improved interpolation technology, which gives an interpolated pixel value toward the minimum value of the block. Then, the difference between the interpolated value and the maximum value (DIM) of the block is applied to embed secret data. Since the gap is enlarged, its embedding capacity also increases. Moreover, the difference expansion (DE) [4,5] is applied to embed a second layer of confidential messages, which further increases the embedding capacity. In 2020, Geetha et al. [21] published a method that uses the last significant bit (LSB) substitution after re-interpolation, resulting in a good visual quality of the confidential images at low payloads. Many other existing RDH methods are also applicable to the interpolation-based RDH scheme.

The scheme published by Mandal et al. [20], in 2021, provides a good embedding capacity compared to other existing schemes. However, when the dynamic extent of pixel values in a block is large, the DE embedding in the second layer leads to severe distortion of the pixel value. We propose an adaptive strategy that first classifies pixel blocks into two types: smooth pixel blocks and complex pixel blocks. Then, different pixel value interpolation and data embedding strategies are designed to fit the feature of the given block. The analysis of the experimental results shows that our proposed strategy is effective both on general images and medical images.

The proposed scheme is elaborated in this paper through the following sections. Some of the related work is briefly described in Section 2. Section 3 presents the proposed adaptive RDH scheme. Section 4 not only provides its own experimental data and compares it with related works but also analyzes the experimental results. Finally, we summarize the proposed scheme in Section 5.

## 2. Related Works

In this section, we introduce two fundamental tools for data hiding, including difference expansion (DE) and the prediction error histogram (PEH), which are used in the related and the proposed RDH schemes. Then, an interpolation-based RDH scheme proposed by Mandal et al. is discussed, which is the main reference of our RDH scheme.

### 2.1. Difference Expansion (DE)

In the differential expansion (DE) [4,5] method, we process the primary image with pixel pairs as the unit of operation. For each pixel pair, the mean value and difference value are calculated first. Centered at the mean value, the difference value is evenly double-expanded outward. We can hide a bit of the confidential message in a pixel pair by switching the difference value between an adjacent pair of even and odd difference values. The formulas for DE are given by:

(1) $$\mu =\lfloor \frac{𝓈+𝓉}{2}\rfloor ,𝒹=\left|𝓈-𝓉\right|,{𝒹}^{\prime }=2\times 𝒹+b.$$

(2) $${𝓈}^{\prime }=\{\begin{array}{c} \mu +\lceil {𝒹}^{\prime }/2\rceil ,\;\;\;\;\;\;\mathrm{if}\;𝓈>𝓉,\\ \mu -\lfloor {𝒹}^{\prime }/2\rfloor ,\;\;\;\;\;\mathrm{otherwise}. \end{array}$$

(3) $${𝓉}^{\prime }=\{\begin{array}{c} \mu -\lfloor {𝒹}^{\prime }/2\rfloor ,\;\;\;\;\;\;\mathrm{if}\;𝓈>𝓉,\\ \mu +\lceil {𝒹}^{\prime }/2\rceil ,\;\;\;\;\;\mathrm{otherwise}. \end{array}$$

The variables (𝓈, 𝓉) denote the cover pixel pair, μ is the average value, 𝒹 is the difference, b is a binary secret bit, ${𝒹}^{\prime }$ is the modified difference, and $\left({𝓈}^{\prime },{𝓉}^{\prime }\right)$ is the marked pixel pair. When we want to extract the information, the secret bit can be read from the new pixel pair by:

(4) $$\mu =\lfloor \frac{{𝓈}^{\prime }+{𝓉}^{\prime }}{2}\rfloor ,{𝒹}^{\prime }=\left|{𝓈}^{\prime }-{𝓉}^{\prime }\right|,\;b=\mathrm{mod}\left({𝒹}^{\prime },2\right),𝒹=\lfloor {𝒹}^{\prime }/2\rfloor .\;𝓈=\{\begin{array}{c} \mu +\lceil 𝒹/2\rceil ,\;\;\mathrm{if}\;{𝓈}^{\prime }>{𝓉}^{\prime },\\ \mu -\lfloor 𝒹/2\rfloor ,\;\;\mathrm{otherwise}. \end{array}\;𝓉=\{\begin{array}{c} \mu -\lfloor 𝒹/2\rfloor ,\;\;\mathrm{if}\;{𝓈}^{\prime }>{𝓉}^{\prime },\\ \mu +\lceil 𝒹/2\rceil ,\;\;\mathrm{otherwise}. \end{array}$$

### 2.2. Prediction Error Histogram (PEH)

**Figure 1:**
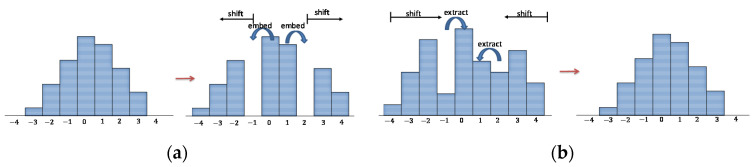
Illustration of data embedding and extraction based on histogram shifting; (**a**) Data embedding process; (**b**) Data extraction process.

There are many PEH-based RDH techniques [8,9,10] and the differences between these algorithms mainly focus on two aspects, which are PEH generation and the PEH modification. For PEH generation, researchers have published various prediction schemes to improve the prediction accuracy and thus generate a more concentrated histogram; for PEH modification, researchers have also proposed many PEH shifting methods to enhance the data embedding rate of confidential images and reduce the expansion distortion. In this paper, we use a commonly applied histogram shifting method to modify PEH. After obtaining the predicted pixel value $D^{\prime }$, we use Equation (5) to calculate the prediction error E and embed the confidential message into the predicted values using the rules given in Equation (6). The corresponding process is illustrated in Figure 1a, where bins 0 and −1 are merged back to bin 0; bins 1 and 2 are merged back to 1; and secret bits are extracted during the process:

(5) $$E=𝒟-D^{\prime },$$

(6) $$D^{\prime \prime }=\{\begin{array}{c} 𝒟+b,\;\;\mathrm{if}\;E=1,\\ 𝒟-b,\;\;\mathrm{if}\;E=0,\\ 𝒟+1,\;\;\mathrm{if}\;E>1,\\ 𝒟-1,\;\;\mathrm{if}\;E<0. \end{array}$$

When we need to extract data, we first obtain the gap $E^{\prime }$ between the marked pixel-value $D^{\prime \prime }$ and the predicted pixel-value $D^{\prime }$ by Equation (7). Then, we can read the confidential message by the rule of Equation (8) while recovering the original gap. The corresponding process is illustrated in Figure 1b, where bins 0 and 1 are selected as the target of embedding; the outside bins are shifted outward to vacate the required embedding space:

(7) $$E^{\prime }=D^{\prime \prime }-D^{\prime },$$

(8) $$D=\{\begin{array}{c} D^{\prime \prime }+1,b=1,\;\;\mathrm{if}\;E^{\prime }=-1,\\ D^{\prime \prime }-1,b=1,\;\;\;\;\;\;\mathrm{if}\;E^{\prime }=2,\\ D^{\prime \prime },b=0,\;\;\;\;\;\;\;\;\;\mathrm{if}\;E^{\prime }=0,\\ D^{\prime \prime },b=0,\;\;\;\;\;\;\;\;\;\mathrm{if}\;E^{\prime }=1,\\ D^{\prime \prime }-1,\;\;\;\;\;\;\;\;\;\;\;\mathrm{if}\;E^{\prime }>2,\\ D^{\prime \prime }+1,\;\;\;\;\;\;\;\;\;\;\mathrm{if}\;E^{\prime }<-1. \end{array}$$

### 2.3. Mandal et al.’s Method [20]

In the method published by Mandal et al. [20], a processing unit of the primary image is a pixel block of size 2×2. Each unit is upsampled into a block of the size 3×3. The inserted pixel values are interpolated using the minimum pixel value and its neighboring pixel values (MN). The data embedding includes two phases. In the first phase, the difference gap between the interpolated pixel value and the maximum pixel value (DIM) is exploited to embed confidential messages with an adjustable length. In the second phase, the interpolated pixels are grouped into two pairs and embedded confidential messages using DE.

**Figure 2:**
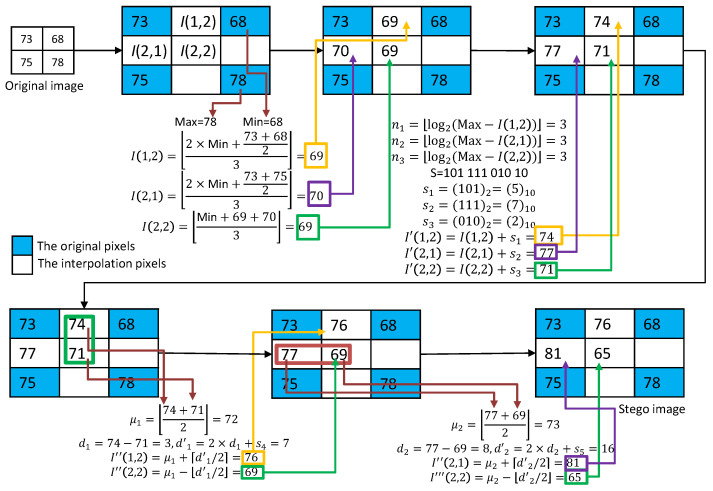
An example of the MN interpolation and the data embedding with the DIM and DE methods.

An example of Mandal et al.’s RDH processing is given in Figure 2**.** As shown in Figure 2, the MN interpolation technique is first applied to calculate the three interpolated pixel values: I(1,2)=69, I(2,1)=70, and I(2,2)=69. In the first embedding phase, DIM is used to estimate the payload of each interpolated pixel. According to the estimated payloads, secret data $s_{1}={\left(101\right)}_{2}$*,* $s_{2}={\left(111\right)}_{2}$, and $s_{3}={\left(010\right)}_{2}$ are embedded to obtain $I^{\prime }\left(1,2\right)=74$, $I^{\prime }\left(2,1\right)=77$, and $I^{\prime }\left(2,2\right)=71$. In the second phase, we first use $I^{\prime }\left(1,2\right)=74$ and $I^{\prime }\left(2,2\right)=71$ as a pair for DE embedding. The secret data $s_{4}=1$ is embedded to obtain $I^{\prime \prime }\left(1,2\right)=76$ and $I^{\prime \prime }\left(2,2\right)=69$. Then, we use $I^{\prime }\left(2,1\right)=77$ and $I^{\prime \prime }\left(2,2\right)=69$ as a pair for DE embedding. The secret data $s_{5}=0$ is embedded to obtain $I^{\prime \prime }\left(2,1\right)=81$ and $I^{\prime \prime \prime }\left(2,2\right)=65$.

In the first phase, DIM in the processing block is exploited to embed data. The larger the difference, the larger the value available for modification, which means that more secret bits can be embedded in the gap. However, the payload would be very small when the neighboring pixel values of a cover image change smoothly. Moreover, DE embedding is applied in the second phase to further improve the ability of the program to embed information. However, the visual quality of the confidential image is greatly degraded when the pixel value difference is large.

In most medical images, the background of the image usually contains a large smooth area, which is not suitable for the application of this RDH method. Moreover, the visual quality of a medical image is a very crucial concern. Therefore, severe degradation after data embedding is not allowed.

## 3. Proposed Scheme

A typical medical image usually contains both a large area of complex textures and a smooth background. For the image blocks with complex textures, we can take advantage of their complexity to embed multiple secret bits as in the DIM method of [20]. However, the additional DE embedding in the second phase results in a large displacement of pixel values, which results in significant visual distortion, so our scheme does not apply an additional DE embedding for such blocks. For the blocks with smooth textures, the payloads of the pixels for the DIM method are usually zero since the pixel values are very close. So, the proposed scheme adopts an alternative interpolation and embedding method to effectively embed secret bits while preserving low distortion.

**Figure 3:**
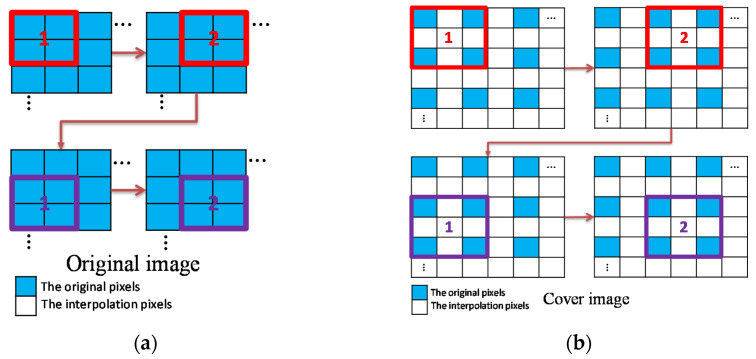
Image dividing, upsampling, and processing order of blocks; (**a**) Image dividing and processing order; (**b**) Processing order of upsampled image blocks.

As illustrated in Figure 3, we follow the interpolation-based framework proposed by Mandal et al. [20] except that the processing order of image blocks is slightly modified. The original medical image is divided into 2×2-sized blocks in an overlapped manner both row-wise and column-wise and the blocks are processed in the raster scanning order.

The dynamic range of pixel values for an image block is defined as the gray level range between the minimum and the maximum pixel values. In order to ensure that the medical images with hidden data have a good visual quality that will not lead to a misdiagnosis, the dynamic range of the pixel values within the processing block is utilized to hide the biometric information. Specifically, the interpolated pixel values modified by the data embedding should not exceed the dynamic range of the original pixel values regardless of whether they are above or below. Otherwise, false noises or contours may occur, and this may lead to misdiagnosis by the physician. However, the dynamic range of the image blocks is not the same. The blocks with a large dynamic range can be used to embed many data bits while the others are not suitable for this treatment. Therefore, we classify the pixel blocks into complex blocks and smooth blocks and process them using different methods.

**Figure 4:**
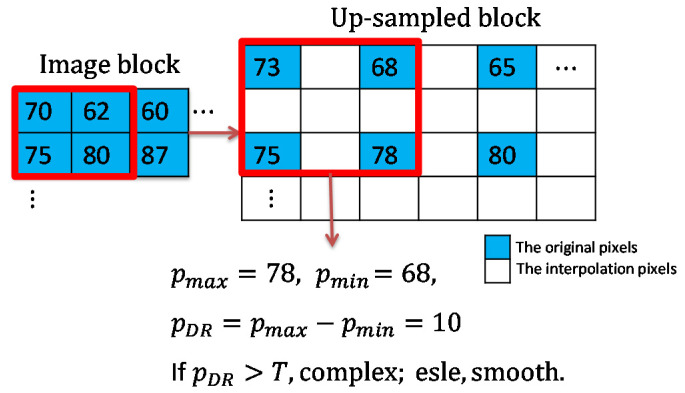
An example of pixel block classification.

**Figure 5:**
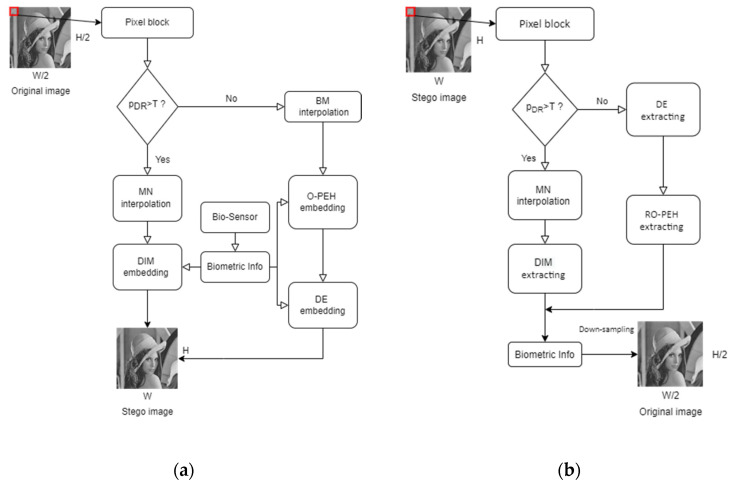
Flowcharts of the embedding and the extraction stages; (**a**) Flowchart of the embedding stage; (**b**) Flowchart of the extraction stage.

The dynamic range of the pixel values in each block is calculated as shown in Figure 4. When the dynamic range of a block is larger than the given threshold, we consider the block to be a complex block; conversely, it is considered to be a smooth block. Meanwhile, the original image is upsampled to obtain a cover image with spaces to be inserted for later use. The detailed processing for image upsampling and block classification is given in Algorithm 1. According to the type of the processing block, a specified interpolation method and embedding method are applied to hide the patient’s biometric information acquired by the biosensors. When a confidential image is received, the receiver first discriminates the block type and extracts the biometric data accordingly. After confirming the patient’s biometric information, the confidential image is downsampled to generate the original image. The overall flowcharts of the embedding and the extraction stages are given in Figure 5.
**Algorithm 1** Image Upsampling and Block ClassificationInput:Original cover image OI-sized ℳ×N, threshold value T.Output: Vacated cover image ℂ-sized (2ℳ−1)×(2N−1).Step 1: Upsample the ℳ×N-sized image OI by zero-interlacing to obtain a (2ℳ−1)×(2N−1))-sized image ℂI, whose values are given by
$\mathbb{C}\left(ℊ,𝒽\right)=\{\begin{array}{c} OI\left(ℊ+1\right)/2,\left(𝒽+1\right)/2),\;ℊ,𝒽\in \mathrm{odd},\\ 0,\;\;\mathrm{otherwise}. \end{array}$      (9) Step 2:Image OI is divided into overlapped 2 × 2 pixel blocks given by
B(𝓈,𝓉)=OI(𝓈:𝓈+1,𝓉:𝓉+1), 1<𝓈<(ℳ−1), 1<𝓉<(N−1).    (10) Step 3: The dynamic range $p_{DR}$ of each block B(𝓈,𝓉) is calculated by
$p_{max}=\max\left\{B\left(𝓈,𝓉\right)\right\}=\max\left\{OI\left(𝓈:𝓈+1,𝓉:𝓉+1\right)\right\},$

$p_{min}=\min\left\{B\left(𝓈,𝓉\right)\right\}=\min\left\{OI\left(𝓈:𝓈+1,𝓉:𝓉+1\right)\right\},$    (11)
$p_{DR}=p_{max}-p_{min}.$  $\mathrm{If}\;p_{DR}>T$, B(𝓈,𝓉) is a complex block; else, it is a smooth block.

### 3.1. Data Embedding Stage

After determining the type of all blocks, all the blocks B(𝓈,𝓉),1<𝓈<(ℳ−1), 1<𝓉<(N−1) are processed in the raster scan order. When the block B(𝓈,𝓉) is to be processed, its corresponding upsampled vacated block ℂ(2𝓈−1:2𝓈+1,2𝓉−1:2𝓉+1) serves as the current processing unit. Depending on the block type of (𝓈,𝓉), two different data embedding processes for complex blocks and smooth blocks are presented as follows.

#### 3.1.1. Data Embedding for Complex Blocks

For complex pixel blocks, the dynamic range of pixel values within the block is relatively large. The data hiding strategy is to embed EPR by filling DIM. To maximize the payload, an interpolated pixel value that is closer to the minimum pixel value of its neighbors is helpful. The MN interpolation method and DIM embedding method proposed by Mandal et al. [20] in 2020 is a successful one. We adopt MN interpolation and DIM embedding, in their first embedding phase, as the embedding method for the complex blocks in our scheme. For the convenience of interpretation, we change the dummy variables in the vacated pixel block ℂ(2𝓈−1:2𝓈+1,2𝓉−1:2𝓉+1) into ℂ(ℊ:ℊ+2,𝒽:𝒽+2). The data embedding process for complex blocks is given in Algorithm 2.
**Algorithm 2** Data Embedding for Complex BlocksInput: Cover block ℂ(ℊ:ℊ+2,𝒽:𝒽+2), secret data S, maximum payload $n_{max}$.Output:Marked block $\hat{\mathbb{C}}\left(ℊ:ℊ+2,𝒽:𝒽+2\right)$.Step 1:Calculate the interpolated pixel values by
$p_{min}=\min\left\{\mathbb{C}\left(ℊ:ℊ+2,𝒽:𝒽+2\right)\right\},$
$\mathbb{C}\left(ℊ,𝒽+1\right)=\left(2\times p_{min}+\left(\mathbb{C}\left(ℊ,𝒽\right)+\mathbb{C}\left(ℊ,𝒽+2\right)\right)/2\right)/3,$

$\mathbb{C}\left(ℊ+1,𝒽\right)=\left(2\times p_{min}+\left(\mathbb{C}\left(ℊ,𝒽\right)+\mathbb{C}\left(ℊ+2,𝒽\right)\right)/2\right)/3,$    (12) $\mathbb{C}\left(ℊ+1,𝒽+1\right)=\left(p_{min}+\mathbb{C}\left(ℊ+1,𝒽\right)+\mathbb{C}\left(ℊ,𝒽+1\right)\right)/3$. Step 2:Calculate the difference values by
$p_{max}=\max\left\{\mathbb{C}\left(ℊ:ℊ+2,𝒽:𝒽+2\right)\right\},{𝒹}_{1}=p_{max}-\mathbb{C}\left(ℊ,𝒽+1\right),$

${𝒹}_{2}=p_{max}-\mathbb{C}\left(ℊ+1,𝒽\right),{𝒹}_{3}=p_{max}-\mathbb{C}\left(ℊ+1,𝒽+1\right).$    (13) Step 3:Calculate the payload for each interpolated pixel by $n_{r}=\min\left(\lfloor \log_{2}\;{𝒹}_{r}\rfloor ,n_{max}\right),r=1,2,3.$    (14) Step 4:Retrieve $n_{1}$$,\;n_{2}$, and $n_{3}$ secret bits from S and convert to decimal values $s_{1}$$,\;s_{2}$, and $s_{3}$. Then, add to the interpolated pixel values as
$\hat{\mathbb{C}}\left(ℊ,𝒽+1\right)=\mathbb{C}\left(ℊ,𝒽+1\right)+s_{1},$

$\hat{\mathbb{C}}\left(ℊ+1,𝒽\right)=\mathbb{C}\left(ℊ+1,𝒽\right)+s_{2},$    (15) $\hat{\mathbb{C}}\left(ℊ+1,𝒽+1\right)=\mathbb{C}\left(ℊ+1,𝒽+1\right)+s_{3}$.

**Figure 6:**
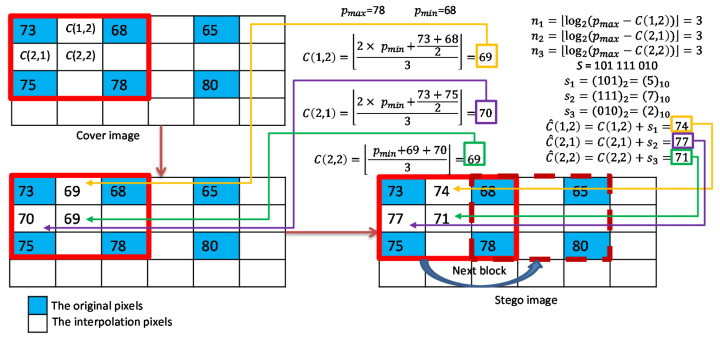
Example of interpolation and data embedding for complex blocks.

As shown in Figure 6, we give an example to illustrate the interpolation and the DIM data hiding strategy of our scheme for a complex block. Three interpolated pixel values ℂ(1,2)=69, ℂ(2,1)=70, and ℂ(2,2)=69 are calculated using MN interpolation. Then, the payloads for each interpolated pixel are calculated. According to the payloads, the data $s_{1}={\left(101\right)}_{2}=5$*,* $s_{2}={\left(111\right)}_{2}=7$*,* and $s_{3}={\left(010\right)}_{2}=2$ are retrieved from the binary secret stream. Finally, the marked pixel values $\hat{\mathbb{C}}\left(1,2\right)=74$*,* $\hat{\mathbb{C}}\left(2,1\right)=77$ and $\hat{\mathbb{C}}\left(2,2\right)=71$ can be calculated.

#### 3.1.2. Data Embedding for Smooth Blocks

For smooth pixel blocks, the dynamic range of pixel values in the block is relatively small. This characteristic provides a very good basis for PEH and DE embedding. Therefore, we design a completely different strategy for pixel value interpolation and data embedding of smooth blocks.

The proposed scheme uses the block mean (BM) as the interpolated pixel values for smooth blocks. Then, the data embedding procedure includes two layers, an ordered PEH (O-PEH) embedding and a DE embedding. For the O-PEH embedding, three interpolated pixels are embedded with secret data in a predefined order. The resulting pixel values are further grouped into two pairs and embedded with secret data using DE.

Since the interpolated pixel values are mutually equal, they are always embeddable, using O-PEH, in the first embedding layer. The resulting pixel values deviate by no more than 2; therefore, the expansion range of DE embedding is also very small. The overall embedding strategy can ensure a good payload and small distortion of the pixel values. The data embedding procedure for smooth blocks is summarized in Algorithm 3.
**Algorithm 3** Data Embedding for Smooth BlocksInput:Cover block ℂ(ℊ:ℊ+2,𝒽:𝒽+2), secret data S.Output:Stego block $\hat{\mathbb{C}}\left(ℊ:ℊ+2,𝒽:𝒽+2\right)$.Step 1:Execute the BM interpolation by$BM=\frac{\mathbb{C}\left(ℊ,𝒽\right)+\mathbb{C}\left(ℊ+2,𝒽\right)+\mathbb{C}\left(ℊ,𝒽+2\right)+\mathbb{C}\left(ℊ+2,𝒽+2\right)}{4}$(16)ℂ(ℊ,𝒽+1)=ℂ(ℊ+1,𝒽)=ℂ(ℊ+1,𝒽+1)=BM.Step 2:Embed layer 1:Embed layer 1: Calculate PEs of the interpolated pixels in the order given by$PE_{1}=\mathbb{C}\left(ℊ+1,𝒽+1\right)-\mathbb{C}\left(ℊ,𝒽+1\right),$ $PE_{2}=\mathbb{C}\left(ℊ,𝒽+1\right)-{\mathbb{C}}^{\prime }\left(ℊ+1,𝒽+1\right),$ $PE_{3}=\mathbb{C}\left(ℊ+1,𝒽\right)-{\mathbb{C}}^{\prime }\left(ℊ+1,𝒽+1\right).$
(17) Embed 1-bit of secret data b from S to the leading pixel in (16) by${\mathbb{C}}^{\prime }=\{\begin{array}{c} \mathbb{C}+b,\;\;\mathrm{if}\;PE=1\\ \mathbb{C}-b,\;\;\mathrm{if}\;PE=0\\ \mathbb{C}+1,\;\;\mathrm{if}\;PE>1\\ \mathbb{C}-1,\;\;\mathrm{if}\;PE<0 \end{array}$(18)Step 3:Embed layer 2:Apply DE to embed 1-bit of secret data from S according to the following processes$\left\{{\mathbb{C}}^{\prime }\left(ℊ,𝒽+1\right),{\mathbb{C}}^{\prime }\left(ℊ+1,𝒽+1\right)\right\}\overset{DE}{\to }\left\{{\mathbb{C}}^{\prime \prime }\left(ℊ,𝒽+1\right),{\mathbb{C}}^{\prime \prime }\left(ℊ+1,𝒽+1\right)\right\},$ $\left\{{\mathbb{C}}^{\prime }\left(ℊ+1,𝒽\right),{\mathbb{C}}^{\prime \prime }\left(ℊ+1,𝒽+1\right)\right\}\overset{DE}{\to }\left\{{\mathbb{C}}^{\prime \prime }\left(ℊ+1,𝒽\right),{\mathbb{C}}^{\prime \prime \prime }\left(ℊ+1,𝒽+1\right)\right\}.$ (19)Step 4:Record ${\mathbb{C}}^{\prime \prime }\left(ℊ,𝒽+1\right),{\mathbb{C}}^{\prime \prime }\left(ℊ+1,𝒽\right),$ and ${\mathbb{C}}^{\prime \prime \prime }\left(ℊ+1,𝒽+1\right)$ as $\hat{\mathbb{C}}\left(ℊ,𝒽+1\right),\hat{\mathbb{C}}\left(ℊ+1,𝒽\right)$ and $\hat{\mathbb{C}}\left(ℊ+1,𝒽+1\right)$, respectively. 

**Figure 7:**
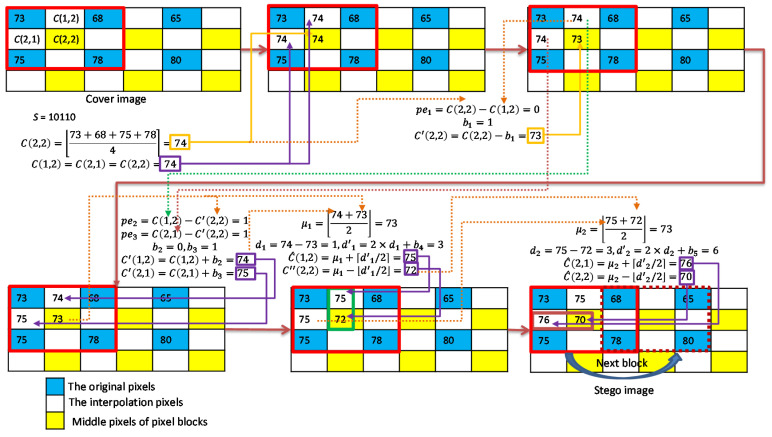
Example of interpolation and data embedding for smooth blocks.

As shown in Figure 7, we give an example to illustrate the BM interpolation and two-layer embedding strategy for smooth pixel blocks. The seed pixels are 73, 68, 75, and 78; and the secret data stream is assumed to be ‘10,110′. We first use the block mean as the interpolated pixel values ℂ(1,2)=ℂ(2,1)=ℂ(2,2)=74. Then, the proposed O-PEH is applied first. The secret data $b_{1}=1$ is embedded to ℂ(2,2) and it results in ${\mathbb{C}}^{\prime }\left(2,2\right)=73$. The next two secret bits $b_{2}=0$ and $b_{3}=1$ are then embedded to and ℂ(2,1), and the resulting pixel values are ${\mathbb{C}}^{\prime }\left(1,2\right)=74$ and ${\mathbb{C}}^{\prime }\left(2,1\right)=75$. In the second embedding layer, we first use ${\mathbb{C}}^{\prime }\left(1,2\right)$ and ${\mathbb{C}}^{\prime }\left(2,2\right)$ as a group for DE embedding and embed secret data $b_{4}=1$, and ${\mathbb{C}}^{\prime \prime }\left(1,2\right)=75$ and ${\mathbb{C}}^{\prime \prime }\left(2,2\right)=72$ are obtained. Finally, ${\mathbb{C}}^{\prime }\left(2,1\right)$ and ${\mathbb{C}}^{\prime \prime }\left(2,2\right)$ are used as a group for DE embedding and to embed secret data $b_{5}=0$, and ${\mathbb{C}}^{\prime \prime }\left(2,1\right)=76$ and ${\mathbb{C}}^{\prime \prime \prime }\left(2,2\right)=70$ are obtained.

### 3.2. Data Extraction Stage

After people receive a stego medical image, the stego medical image is first divided into pixel blocks sized 3×3 that overlap between rows and columns with a stride of 2 to ensure that the processing units are exactly the same as the embedding. Then, the dynamic range of pixel values for each block is calculated to classify the blocks into complex and smooth types. The data extraction algorithms for complex and smooth blocks are described in the following subsections. After extracting the secret data, the confidential image is downsampled to obtain the original medical image.

#### 3.2.1. Data Extraction for Complex Pixel Blocks

For a complex pixel block, we first calculate the interpolated pixel values using the seed pixel values so that we can obtain the gap between each original interpolated pixel value and the maximum value. The payload for each interpolated pixel is therefore obtained. Then, we obtain our secret data by converting the difference between the confidential pixel value and the maximum value back to the binary secret bits with the length determined by its payload. The extraction procedure of complex blocks is summarized in Algorithm 4.

**Figure 8:**
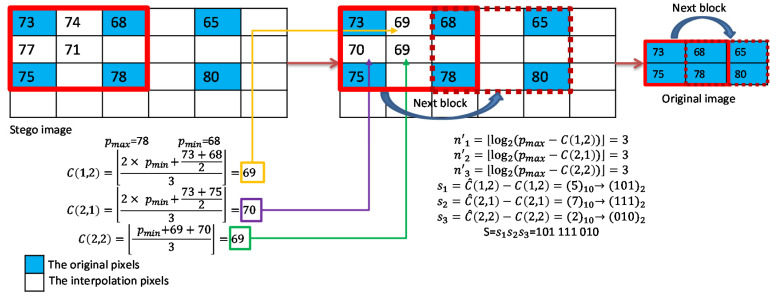
Example of data extraction for complex blocks.

An example of data extraction for complex blocks is shown in Figure 8. The original interpolated pixel values ℂ(1,2)=69, ℂ(2,1)=70, and ℂ(2,2)=69 are calculated first. Then, the difference values with respect to $p_{max}=78$ are calculated to determine the payloads. Finally, the difference between each stego pixel value and original interpolated value is calculated and converted to binary bits according to its payload as shown in the figure.
**Algorithm 4** Data Extraction for Complex BlocksInput: Stego block $\hat{\mathbb{C}}\left(ℊ:ℊ+2,𝒽:𝒽+2\right)$, maximum payload $n_{max}$.Output: Secret data S.Step 1:Calculate the original interpolated pixel values by
$\mathbb{C}\left(ℊ,𝒽+1\right)=\left(2\times p_{min}+\left(\hat{\mathbb{C}}\left(ℊ,𝒽\right)+\hat{\mathbb{C}}\left(ℊ,𝒽+2\right)\right)/2\right)/3,$
$\mathbb{C}\left(ℊ+1,𝒽\right)=\left(2\times p_{min}+\left(\hat{\mathbb{C}}\left(ℊ,𝒽\right)+\hat{\mathbb{C}}\left(ℊ+2,𝒽\right)\right)/2\right)/3,$    (20) $\mathbb{C}\left(ℊ+1,𝒽+1\right)=\left(p_{min}+\hat{\mathbb{C}}\left(ℊ+1,𝒽\right)+\hat{\mathbb{C}}\left(ℊ,𝒽+1\right)\right)/3$. Step 2:Calculate the difference values by Step 3:$d_{1}=p_{max}-\mathbb{C}\left(ℊ,𝒽+1\right),$ $d_{2}=p_{max}-\mathbb{C}\left(ℊ+1,𝒽\right),$   (21) $d_{3}=p_{max}-\mathbb{C}\left(ℊ+1,𝒽+1\right).$
Calculate the payload of each interpolated pixel by
$n_{r}=\min\left(\lfloor \log_{2}d_{r}\rfloor ,n_{max}\right),\;r=1,2,3.$    (22) Step 4:Calculate the difference values and restore secret bits according to $n_{r}$. $s_{1}=\hat{\mathbb{C}}\left(ℊ,𝒽+1\right)-\mathbb{C}\left(ℊ,𝒽+1\right),$
$s_{2}=\hat{\mathbb{C}}\left(ℊ+1,𝒽\right)-\mathbb{C}\left(ℊ+1,𝒽\right),$    (23) 
$s_{3}=\hat{\mathbb{C}}\left(ℊ+1,𝒽+1\right)-\mathbb{C}\left(ℊ+1,𝒽+1\right).$


#### 3.2.2. Data Extraction for Smooth Pixel Blocks

For a smooth pixel block, the data extraction operation is executed according to the reverse embedding order of the pixel block. We first restore the secret bits hidden in the second layer. In this layer, the second pair of pixels is processed and then the first pair. Finally, the secret bits hidden in the first layer are also extracted in the reverse order of PEH (RO-PEH). Algorithm 5 summarizes the data extraction process of the smooth block.
**Algorithm 5** Data Extraction for Smooth BlocksInput:Stego block $\hat{\mathbb{C}}\left(ℊ:ℊ+2,𝒽:𝒽+2\right)$.Output:Secret data S.
Extract layer 2:Step 1:Apply RDE, (4), to extract secret data according to the following steps$\left\{\hat{\mathbb{C}}\left(ℊ+1,𝒽\right),\hat{\mathbb{C}}\left(ℊ+1,𝒽+1\right)\right\}\overset{RDE}{\to }\left\{{\mathbb{C}}^{\prime }\left(ℊ+1,𝒽\right),{\mathbb{C}}^{\prime }\left(ℊ+1,𝒽+1\right)\right\},$ $\left\{\hat{\mathbb{C}}\left(ℊ,𝒽+1\right),{\mathbb{C}}^{\prime }\left(ℊ+1,𝒽+1\right)\right\}\overset{RDE}{\to }\left\{{\mathbb{C}}^{\prime }\left(ℊ,𝒽+1\right),{\mathbb{C}}^{\prime \prime }\left(ℊ+1,𝒽+1\right)\right\}.$(24)
*Extract layer 1*:Step 2:Apply RPEH, Equations (7) and (8), to extract secret data according to the following steps$\left\{{\mathbb{C}}^{\prime }\left(ℊ+1,𝒽\right),{\mathbb{C}}^{\prime \prime }\left(ℊ+1,𝒽+1\right)\right\}\overset{RPEH}{\to }\left\{{\mathbb{C}}^{\prime \prime }\left(ℊ+1,𝒽\right),{\mathbb{C}}^{\prime \prime }\left(ℊ+1,𝒽+1\right)\right\},$ $\left\{{\mathbb{C}}^{\prime }\left(ℊ,𝒽+1\right),{\mathbb{C}}^{\prime \prime }\left(ℊ+1,𝒽+1\right)\right\}\overset{RPEH}{\to }\left\{{\mathbb{C}}^{\prime \prime }\left(ℊ,𝒽+1\right),{\mathbb{C}}^{\prime \prime }\left(ℊ+1,𝒽+1\right)\right\},$ $\left\{{\mathbb{C}}^{\prime \prime }\left(ℊ+1,𝒽+1\right),{\mathbb{C}}^{\prime \prime }\left(ℊ,𝒽+1\right)\right\}\overset{RPEH}{\to }\left\{{\mathbb{C}}^{\prime \prime \prime }\left(ℊ+1,𝒽+1\right),{\mathbb{C}}^{\prime \prime }\left(ℊ,𝒽+1\right)\right\}.$(25)

**Figure 9:**
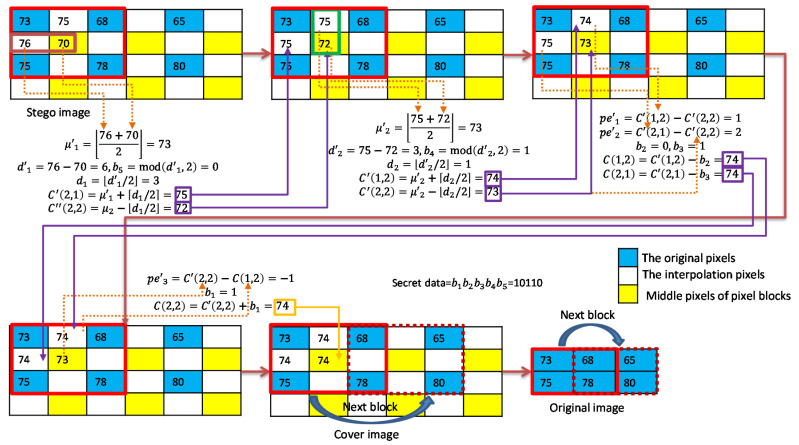
Example of data extraction for smooth blocks.

An example of data extraction for smooth blocks is shown in Figure 9. In the extraction of the second layer, the stego pixel pair $\left\{\hat{\mathbb{C}}\left(2,1\right)=76,\;\hat{\mathbb{C}}\left(2,2\right)=70\right\}$ is applied to extract $b_{5}=0$ and recover to $\left\{{\mathbb{C}}^{\prime }\left(2,1\right)=75,{\mathbb{C}}^{\prime }\left(2,2\right)=72\right\}$. Then, the pixel pair $\left\{\hat{\mathbb{C}}\left(1,2\right)=75,{\mathbb{C}}^{\prime }\left(2,2\right)=72\right\}$ is applied to extract $b_{4}=1$ and recover to $\left\{{\mathbb{C}}^{\prime }\left(1,2\right)=74,{\mathbb{C}}^{\prime \prime }\left(2,2\right)=73\right\}$. The extraction of the first layer is processed as follows. The prediction errors of pixel ${\mathbb{C}}^{\prime }\left(2,1\right)=75$ and ${\mathbb{C}}^{\prime }\left(1,2\right)=74$ with respect to ${\mathbb{C}}^{\prime \prime }\left(2,2\right)=73$ are calculated to extract $b_{3}=1,b_{2}=0$ and recover to ${\mathbb{C}}^{\prime \prime }\left(2,1\right)=74,{\mathbb{C}}^{\prime \prime }\left(1,2\right)=74$. Finally, the prediction error of pixel ${\mathbb{C}}^{\prime \prime }\left(2,2\right)=73$ with respect to ${\mathbb{C}}^{\prime \prime }\left(1,2\right)=74$ is calculated to extract $b_{1}=1$ and recover to ${\mathbb{C}}^{\prime \prime }\left(2,2\right)=74$.

### 3.3. Overflow and Underflow

In the process of embedding secret information, extreme pixel values may cause overflow or underflow problems. In the embedding process of a complex block, pixel values are always modified within the dynamic range of the seed pixel values. Therefore, there is no overflow/underflow problem.

The smooth pixel blocks of the block mean valued 0 and 255 are left unembedded to prevent overflow/underflow of the first-layer embedding while the smooth pixel blocks of the block mean valued within the ranges 1 to 3 and 253 to 254 are only processed with the first-layer embedding to prevent the overflow/underflow of the second-layer embedding.

## 4. Experimental Results and Discussion

**Figure 10:**
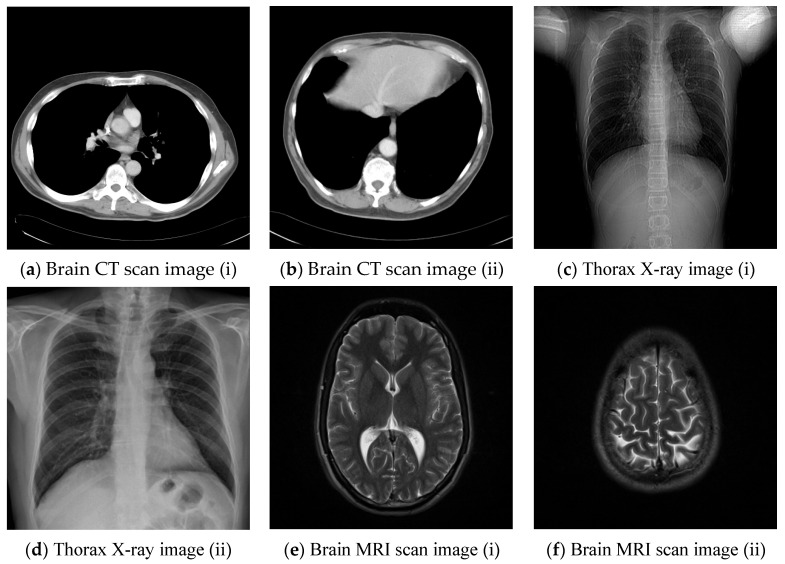
Six medical test images; (**a**) Brain CT scan image (i); (**b**) Brain CT scan image (ii); (**c**) Thorax X-ray image (i); (**d**) Thorax X-ray image (ii); (**e**) Brain MRI scan image (i); (**f**) Brain MRI scan image (ii).

Our proposed adaptive RDH scheme was implemented on the Windows PC operating system using MATLAB version R2017a. We first verified the feasibility of our proposed adaptive RDH scheme with nine standard grayscale test images sized 512×512, and then tested it on the six medical images shown in Figure 10. We regarded the binary secret data S produced by a random number generator as the EPR to be embedded in the image. The threshold for block type classification was set to 5. The performance of the adaptive RDH scheme was evaluated with the peak signal to noise ratio (PSNR), the structural similarity (SSIM), and the embedding capability (EC). PSNR is a metric used to measure the visual quality of confidential images. Here, we adopted the PSNR definition in [2] as follows:

(26) $$\mathrm{PSNR}=10\log_{10}\left(\frac{H\times W\times {\left(255\right)}^{2}}{\sum_{i=1}^{H}\sum_{j=1}^{W}{\left(G_{ij}-{\hat{G}}_{ij}\right)}^{2}}\right),$$

where H×W is the size of the cover image, and $G_{ij}$ and ${\hat{G}}_{ij}$ denote the pixel values of the cover image and the stego-image, respectively. To know whether the confidential image is close to a natural image or not, we first downsample a test image to obtain the small image. Then, it is processed by the proposed scheme to obtain a confidential image. Finally, the PSNR value of the confidential image is measured according to the given test image. Through the experiments, if the PSNR value is larger than 30 dB, it means that the distortion caused by information embedding is not detectable by the human eye. Of course, the higher the PSNR value, the less distortion due to hidden data. By minimizing the alteration of the pixel values, confidential images with a high PSNR value can be obtained.

SSIM is also a metric used to evaluate the similarity between the original image and the stego-image, as shown in Equation (27):

(27) $$\mathrm{SSIM}=\frac{\left(2\mu_{x}\mu_{y}+c_{1}\right)\left(2\sigma_{xy}+c_{2}\right)}{\left(\mu_{x}{}^{2}+\mu_{y}{}^{2}+c_{1}\right)\left(\sigma_{x}{}^{2}+\sigma_{y}{}^{2}+c_{2}\right)}$$

where $\mu_{x}$ and $\mu_{y}$ denote the mean of images x and y, respectively; $\sigma_{x}$, $\sigma_{y}$, $\sigma_{xy}$ denote the standard deviation and covariance of images x and y, respectively; and $c_{1}$ and $c_{2}$ are two constants to avoid division by zero. The SSIM metric takes into account both image luminance, contrast, and structure information, which is closer to the benchmark of the human visual system; the higher the SSIM value, the more similar the original image and the stego image are.

EC, measured in bits per pixel, is defined as the total amount of payload in bits divided by the total amount of pixels in the interpolated cover image.

### 4.1. Experimental Results for Standard Grayscale Images

Our proposed adaptive RDH scheme was compared with the interpolation-based RDH schemes, proposed by Mandal et al. [20] and Geetha et al. [21], using the standard grayscale images. The values of EC, PSNR, and SSIM for the compared schemes are listed in Table 1. Since the scheme proposed by Geetha et al. [21] uses LSB substitution, its embedding capability is a fixed value and the features of the pixel blocks are not taken into consideration. In comparison with [21], our scheme provides a better EC with a comparable SSIM. Although the PSNR value of our scheme is lower than that of [21], the distortion is imperceptible when its value is higher than 30 dB. Mandal et al.’s scheme [20] provides the highest EC among the three schemes; however, its PSNR and SSIM are the worst. Our proposed adaptive RDH scheme has a good balance between PSNR and EC by taking the block feature into consideration. Numerically, the PSNR values obtained by our proposed adaptive RDH scheme are all greater than 30 dB, which means that the difference between the confidential image obtained by the proposed scheme and the original test image is relatively small, and the confidential image looks like a natural one.

### 4.2. Experimental Results for Medical Images

**Figure 11:**
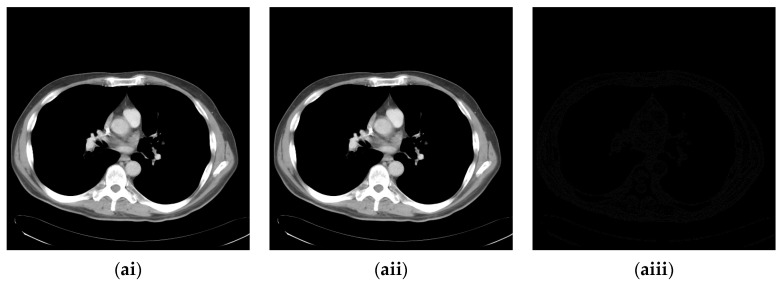

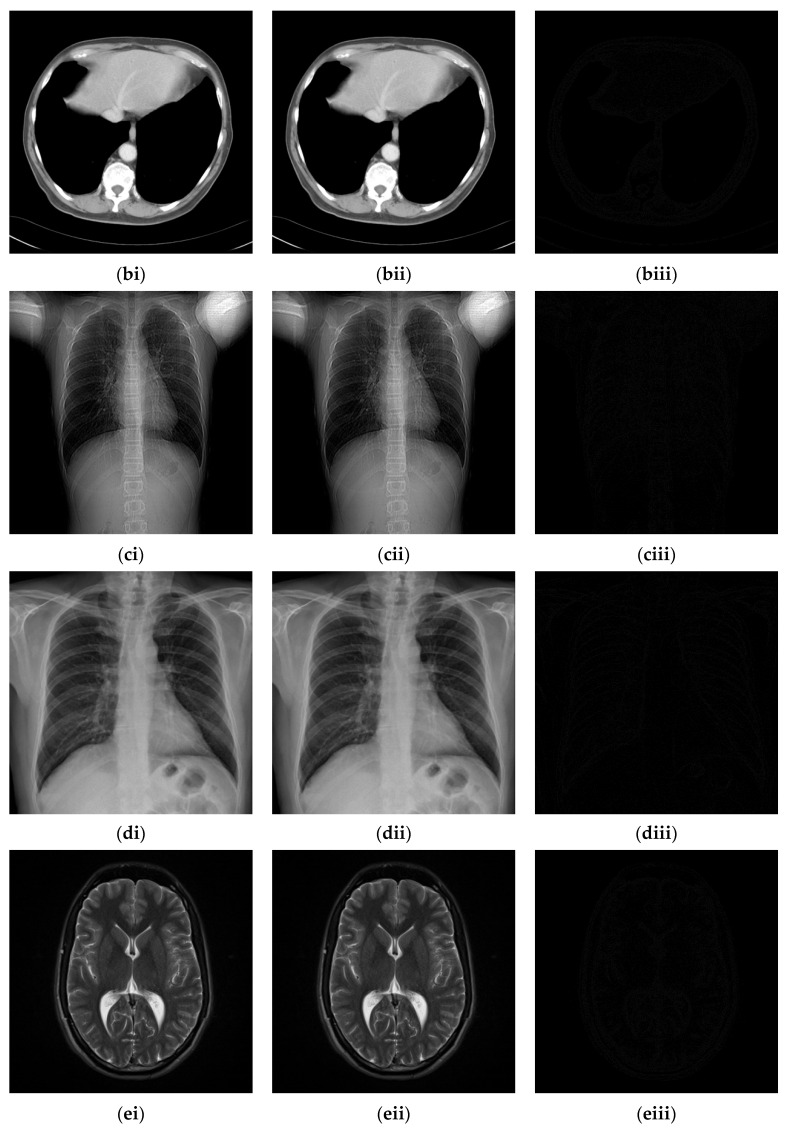

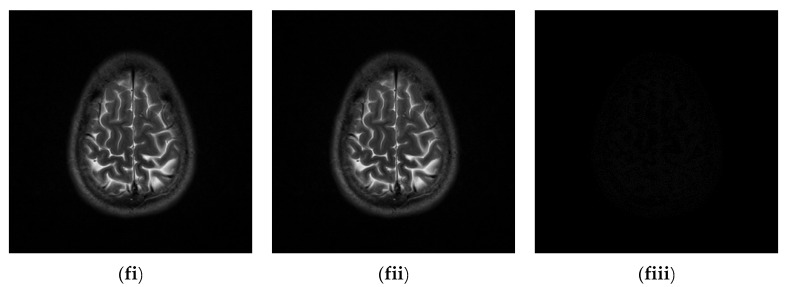
The original images, the confidential images, and the different images; (**ai**–**fi**) The cover images; (**aii**–**fii**) The confidential images; (**aiii**–**fiii**) The difference between the cover images and the confidential images.

Since the interpolation-based RDH schemes are designed for the special purpose of embedding patient information into medical images, the performance of our proposed adaptive RDH scheme on medical images is the main concern. Figure 11 shows the original image and its corresponding confidential image with 2 bpp secret data embedded, and images showing the difference between the original medical images and their confidential images. The difference images (aiii–fiii) in Figure 11 show almost nothing, which indicates that the proposed scheme does not look much different from the original image after hiding and will not draw the eavesdropper’s attention.

Table 2 shows the PSNR values of our proposed adaptive RDH scheme for the medical images in Figure 10, where the embedding rate ranges from 0.25 to 2 bpp. From the table, we can clearly see that PSNR of the proposed scheme reaches 53.3405 dB in a low embedding rate, which indicates that our proposed adaptive RDH scheme does not cause significant distortion to the images. In a high embedding rate, PSNR of our proposed adaptive RDH scheme is still more than 36 dB.

A comparison of the proposed scheme with related works is given in Table 3, where p and q represent the number of bits that can be embedded in edge pixels and non-edge pixels in [27], respectively, and k is the number of bits per embedding in [30]. From the table, we can find that our proposed adaptive RDH scheme significantly outperforms most of the related works in terms of both EC and PSNR, which means that our scheme can hide more biometric information while preserving a better visual quality than the others. For [19,26,29], our scheme wins over these schemes both in terms of EC and PSNR. In [27], when p=4,q=3, EC of [27] is similar to that of our proposed adaptive RDH scheme, but PSNR of [27] is worse than that of our proposed adaptive RDH scheme. When p=4,q=4, EC of [27] can reach 3 bpp and PSNR of the image is obviously much worse. While the proposed scheme is slightly inferior to [27] in terms of EC, we are able to maintain PSNR of the image at a relatively good level. In [30], when k=2, the scheme loses to the proposed scheme in both EC and PSNR. When k=3, although the proposed scheme is slightly inferior to [30] in terms of EC, the higher value of PSNR of our steganographic image makes the image carrying information less detectable during transmission.

**Figure 12:**
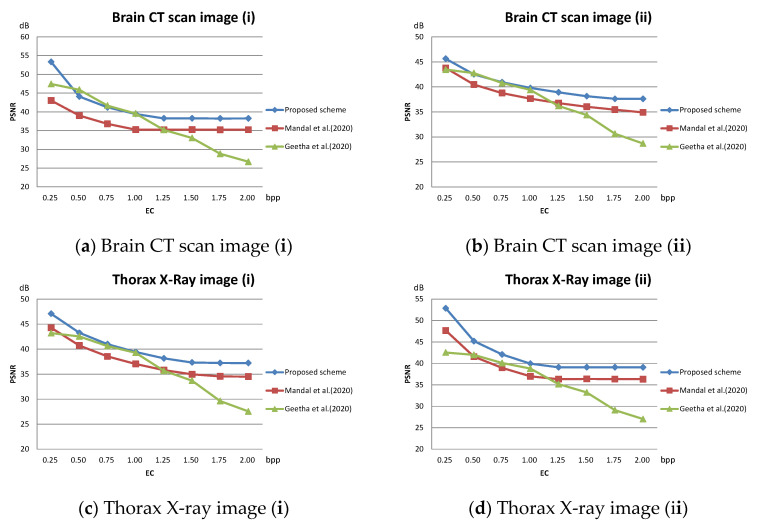

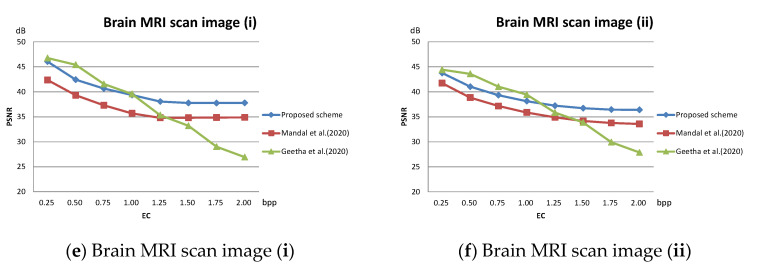
Comparison of PSNR on medical images; (**a**) Brain CT scan image (i); (**b**) Brain CT scan image (ii); (**c**) Thorax X-ray image (i); (**d**) Thorax X-ray image (ii); (**e**) Brain MRI scan image (i); (**f**) Brain MRI scan image (ii).

The PSNR value with respect to the payload of three related schemes for the test images given in Figure 10 are plotted in Figure 12. As shown in the figures, our scheme maintains a superior visual quality under an equal payload. When the payload is larger than 1.5 bpp, the PSNR value of Geetha et al.’s scheme [21] degrades significantly.

The method published by Mandal et al. [20] treats all pixel blocks the same. When executing DE embedding in its second data hiding phase, the large pixel value differences cause the pixel values to be drastically modified, which significantly reduces the PSNR value of the stego image. In our scheme, the pixel blocks are classified into smooth and complex ones in advance. During data hiding, DE embedding of complex blocks is omitted, which effectively improves the PSNR value. For the smooth blocks, the block mean interpolation ensures a controllable difference value between interpolated pixels and DE can be safely executed without leading to significant distortion. The experimental data confirms the effectiveness of the proposed strategy. With this strategy, our scheme is not only able to achieve good visual quality scores on general grayscale images but is also able to embed more secret messages on medical images. The proposed scheme provides a high PSNR value at a low hiding volume. As the embedded volume increases, PSNR of the proposed scheme does not decrease drastically as in [21] but decreases smoothly.

## 5. Conclusions

A new interpolation-based RDH scheme was proposed for hiding patient biometric information in medical images. The pixel blocks were classified into complex and smooth ones. Different methods were applied to process the two different categories. For complex blocks, the dynamic range of pixel values were preserved during data embedding. For smooth blocks, two layers of an electronic patient record can be embedded with a slight distortion in the pixel values. Compared to [20], our adaptive scheme uses the same MN interpolation and DIM embedding to ensure a high payload for the image blocks with complex textures but removes the DE embedding part of [20], which causes high distortion for the complex blocks. For the smooth image blocks, MN interpolation and DIM embedding methods cannot provide a satisfactory payload, so we adopted BM interpolation and DE embedding to ensure a good payload while preserving low distortion. From the experimental results, we can conclude that our proposed adaptive scheme can embed a satisfactory amount of biometric information with the least degree of image distortion. Our proposed adaptive scheme was also compared with state-of-the-art schemes. The performance improvement confirms the excellence of our proposed adaptive scheme.

In the application of medical image management, when the embedding capacity is large enough, the extra space can be applied to embed electronic patient records. In the future, we will focus on designing an RDH scheme that combines the hiding of patient biometric information and electronic diagnosis records.

## Figures and Tables

**Table 1:** Comparison of PSNR, SSIM and EC with related works.

Images	Proposed Scheme			[20]			[21]		
	EC	PSNR	SSIM	EC	PSNR	SSIM	EC	PSNR	SSIM
Airplane	1.93	35.30	0.95	2.17	33.23	0.92	1.50	39.46	0.94
Baboon	2.73	31.57	0.94	3.19	29.40	0.86	1.50	39.44	0.97
Boat	2.30	33.68	0.94	2.70	31.65	0.86	1.50	39.43	0.95
Couple	2.33	33.49	0.94	2.56	32.05	0.87	1.50	39.41	0.95
Elaine	2.29	34.06	0.92	2.50	33.10	0.83	1.50	39.45	0.94
Goldhill	2.32	33.56	0.93	2.72	31.62	0.84	1.50	39.43	0.95
Lena	2.05	35.00	0.94	2.39	33.03	0.88	1.50	39.44	0.94
Man	2.35	33.22	0.92	2.73	31.15	0.85	1.50	39.49	0.95
Peppers	2.06	35.26	0.94	2.42	33.13	0.88	1.50	39.42	0.94

**Table 2:** PSNR for different EC values.

	EC	0.25 bpp	0.50 bpp	0.75 bpp	1.00 bpp	1.25 bpp	1.50 bpp	1.75 bpp	2.00 bpp
Images									
CT (i)		53.3405	44.0953	41.1997	39.4263	38.2624	38.2767	38.2376	38.2550
CT (ii)		45.6208	42.5201	40.9425	39.7861	38.9088	38.1414	37.6155	37.5999
X-ray (i)		47.0919	43.2664	40.9943	39.4476	38.1728	37.3442	37.2253	37.2143
X-ray (ii)		52.8555	45.1903	42.0803	39.9389	39.0696	39.1002	39.1011	39.0826
MRI (i)		46.0622	42.4452	40.6458	39.3618	38.0475	37.7737	37.7601	37.7896
MRI (ii)		43.7435	41.0151	39.3316	38.1186	37.2286	36.7140	36.4273	36.3968

**Table 3:** Comparison of PSNR and EC with related works.

Images	Proposed		[19]		[26]		[27] (*p* = 4, q = 3)		[27] (*p* = 4, q = 4)		[29]		[30] (k = 2)		[30] (k = 3)	
	EC	PSNR	EC	PSNR	EC	PSNR	EC	PSNR	EC	PSNR	EC	PSNR	EC	PSNR	EC	PSNR
Airplane	1.93	35.30	1.34	22.91	0.87	27.37	2.33	33.06	3.00	30.85	1.5	26.47	1.48	32.08	2.95	31.92
Baboon	2.73	31.57	2.27	20.49	1.75	21.22	2.41	29.60	3.00	28.58	1.5	25.55	\	\	\	\
Lena	2.05	35.00	1.32	22.32	0.86	30.24	2.33	34.40	3.00	31.56	1.5	26.85	1.48	33.45	2.95	33.23
Peppers	2.06	35.26	1.29	22.45	0.85	28.04	2.32	33.66	3.00	31.17	\	\	1.48	31.00	2.20	30.88

## Data Availability

Not applicable.
